# The new WHO classification for essential thrombocythemia calls for revision of available evidences

**DOI:** 10.1038/s41408-020-0290-9

**Published:** 2020-02-25

**Authors:** Tiziano Barbui, Jürgen Thiele, Alberto Ferrari, Alessandro M. Vannucchi, Ayalew Tefferi

**Affiliations:** 1 FROM Research Foundation, Papa Giovanni XXIII Hospital, Bergamo, Italy; 2 Institute of Pathology, University of Cologne, Cologne, Germany; 3 CRIMM-Center of Research and Innovation of Myeloproliferative Neoplasms, Azienda Ospedaliera Universitaria Careggi, Department Experimental and Clinical medicine, and Denothe Center, University of Florence, Florence, Italy; 4 Division of Hematology, Department of Internal Medicine, Mayo Clinic, Rochester, MN USA

**Keywords:** Myeloproliferative disease, Diagnosis

## Abstract

In the 2016 revised classification of myeloproliferative neoplasms pre-fibrotic primary myelofibrosis (pre-PMF) was recognized as a separate entity, distinct from essential thrombocythemia (ET). Owing that the majority of cases falling in the pre-PMF category were previously diagnosed as ET, one may question about the need to re-evaluate the results of epidemiologic, clinical, and molecular studies, and the results of clinical trials in the two entities. Based on a critical review of recently published studies, pre-PMF usually presents with a distinct clinical and hematological presentation and higher frequency of constitutional symptoms. *JAK2V617F* and *CALR* mutations in pre-PMF patients are superimposable to ET, whereas non-driver high-risk mutations are enriched in pre-PMF compared with ET. Thrombosis is not significantly different, whereas bleeding is more frequent in pre-PMF. Median survival is significantly shorter in pre-PMF and 10-year cumulative rates progression to overt myelofibrosis is 0–1% vs. 10–12%, and leukemic transformation is 1–2% vs. 2–6%, in ET and pre-fibrotic-PMF, respectively. Most patients fall in the lower prognostic IPSS group in which observation alone can be recommended. Patients at intermediate risk may require a symptom-driven treatment for anemia, splenomegaly or constitutional symptoms while cytoreductive drugs are indicated in the high-risk category.

## Introduction

Compared with the 2008 WHO diagnostic criteria for essential thrombocythemia (ET)^1^ in the 2016 revised version^2,3^ diagnostic features of pre-fibrotic primary myelofibrosis (pre-PMF) were stringently defined and this entity was recognized as a separate category, distinct from “true” ET^4–10^, mainly based on scrutinized evaluation of bone marrow (BM) biopsy features.^11–13^. This was a major advancement that significantly modified the diagnostic landscape and deserves practical implications.^10^ Furthermore, owing that the majority of cases currently falling in the pre-PMF category were diagnosed as ET in the past, one may question about the need to critically re-evaluate the results of epidemiologic, clinical, and molecular studies, as well as the results of clinical trials,^14^ in the context of the two entities.

In this work, we collected and reviewed the most-relevant information on this matter, aiming at providing a comprehensive evaluation of the issue at hand.

## Differences in epidemiology of ET

### Incidence

The true overall incidence of ET is not known as available data are based on estimates derived from patients diagnosed according to the guidelines of the Polycythemia Vera Study Group (PVSG) criteria that do not differentiate between “true” ET and pre-PMF.^15^ Regarding the well-documented registries of Sweden a population-based retrospective survey was conducted in the city of Götenborg covering the years 1983–1999 (i.e., before the WHO classification) revealing an annual of incidence 1.55 per 100,000 inhabitants for ET vs. 1.97 per 100,000 inhabitants for polycythemia vera (PV).^16^ According to the Swedish National MPN Registry and the 2008 WHO diagnostic guidelines^1^ during the period 2008 to 2015 the corresponding data of 1284 newly diagnosed ET patients increased to 2.0 per 100,000 and of the 1105 PV patients incidence was reduced to 1.8 per 100,000.^17^ Dynamics of these epidemiological data may be influenced by a number of points: (1) there are no data referring to pre-PMF or a reclassification of the ET cohort, (2) regarding PV significant lowering of the hemoglobin/hematocrit diagnostic threshold values in the 2016 WHO revision^2,3^ were not regarded, (3) application of mutation analysis as important tool for diagnosis, and (4) increase in automatic blood sample analysis may both have raised the possibility to diagnose MPNs at a very early stage.

On the other hand, the relative incidence of pre-PMF may be grossly estimated by considering results obtained during a reclassification of representative BM biopsies from treatment-naive so-called ET patients to tackle “true” ET cases^18,19^. By following this procedure in a cohort of 404 patients recruited between 1983 and 2005 a reclassification of the 358 “old” ET cases according to 2016 WHO criteria^2^, 268 (75%) were reclassified as ET, 25 (7%) as unclassifiable and 65 (18%) as pre-PMF^5^. Other groups reported rates of 16^20^, 17.6^10^, or 14%^8^ of patients with pre-PMF in their so-called ET cohorts after re-evaluation by strictly applying the WHO diagnostic criteria^2,3^. In this context, one should be aware that these data are certainly representing the lower ranges as derived from centers of excellence. The extremely high incidence of 51% pre-PMF cases reported by Rupoli et al.^21^ was based on the failure to regard only BM specimens with fiber grade 0 (normal) and missing fiber grade 1 (minor)^22,23^ as requested by the WHO classification.^1,18^ In clinical practice, a rate of pre-PMF mimicking ET ranging between 20 and 30% may be closer to reality. Consequently, provided pre-PMF is definitely excluded “true” ET may become the rarest subtype of major MPNs (except for MPN-unclassifiable). At least according to the “real world” concerning presenting patients the strictly WHO 2016-defined Austrian Reclassification Project including presently 807 MPNs lists as most frequent subtype PV with 44.7% compared with 27.4% with ET.^10^

### Presentation of clinical data

At diagnosis a number of clinical characteristics and hematological parameters reveal significant differences when comparing patients with pre-PMF and ET.^19^

Patients with pre-PMF usually present with higher counts for leukocytes, lower hemoglobin levels, higher LDH values, and more frequently palpable splenomegaly.^5,12,18,20^ Increase in circulating CD 34-positive progenitor cells has been also reported.^4,20^ Constitutional symptoms (night sweats, fatigue, weight loss) have been reported in 20.5% of pre-PMF patients^4^ compared with 14.8% in ET.^12^ Moreover, only rarely a very few erythroblasts and/or myeloblasts (<2%) may be detectable in peripheral blood smears (left shifting) and no leukoerythroblastosis in both subtypes.^18^

Changes concerning molecular findings are interesting, particularly if performed by using reclassified material taking explicitly pre-PMF into account.^24^ Incidences of *JAK2*V617F mutations are very similar in ET and pre-PMF ranging between 54% and 66%^5,19,20,25,26^ and 52% and 67%^4,5,19,20^, respectively. Calreticulin (*CALR*: 19p13.2) mutations may be found in 15–24% in ET patients^25,26^ with a tendency to increase in pre-PMF.^5,19^ *CARL* mutations in ET showed good predictability of survival in pre-PMF but not ET, with *CALR* being a more favorable mutation than *JAK2*.^19^ This finding confirms and extends data from a multicenter study on overt PMF.^25^ On the other hand, according to multivariable analysis even after *CARL* variant stratification it has been questioned that driver mutations might influence outcome.^27^ Most interesting is that in this context *JAK2/MPL* mutations were associated with a higher risk of fibrotic progression in ET.^28^ These observations are supporting the results reported recently by Szuber, N. et al.^29^ on reclassified BM samples by suggesting the possibility that a number of *MPL*-mutated ET might actually represent pre-fibrotic PMF. It has to be noted that *MPL* (myeloproliferative leukemia virus oncogene; 1p34) mutations are rare and occur in <4% of ET patients^5,24,30^, ~6% in pre-PMF^4,5^ and 6–8% of overtly fibrotic PMF patients.^4,12^ M*PL*-mutated ET cohorts have higher reported rates of fibrotic progression than their *MPL* wild-type counterparts with 33.3% (vs. 7.5% in *MPL*-unmutated) in some series.^25,30^ The 776 ET patients from the UK-PT1 Study showed a high *MPL* mutation rate of 8.5%^31^, which is not surprising as this cohort was diagnosed according to the PVSG criteria^15^ that allow in contrast to the WHO guidelines^1–3^ a certain degree of overt myelofibrosis (MF) at onset, suggesting rather pre-PMF than “true” ET. It has been postulated based on reclassification experience that the majority of routinely assigned cases of MPL-mutated ET probably represent pre-fibrotic PMF when morphologically scrutinized.^29^

## Differences in outcomes and prognosis

The revised 2016 WHO classification system distinguishes “pre-fibrotic” from “overtly fibrotic” PMF; the former might mimic ET in its clinical presentation and therefore, it is prognostically relevant to distinguish the two entities.^20,32^ Generally, as the other MPNs the clinical course of ET and pre-PMF is characterized by vascular events and an inherent tendency to progress into overt MF and blast phase (BP).^5,20^ The salient problem we are facing is that clinical findings and outcome are significantly depending on the classification system that was applied including PVSG or WHO criteria or a mixture of both, especially in retrospective studies including older ET cases from the files without critical review or patients with previous therapy. These difficulties have been outlined in a review article reporting a very large cohort of ET patients with long-term outcome regarding wide ranges of overall survival (11–22.6 years), transformation to BP (6.3–14.5 median time in years), and MF (7.3–16 median time in years).^14^

### Vascular events

Thrombosis and hemorrhage represent two of the main causes of morbidity and mortality in patients with ET. Incidence of arterial and venous thrombosis prior to diagnosis revealed no significant differences (23% /20 and 9/8%) in WHO-defined ET compared with pre-PMF^33^; thrombotic complications were also similar during the follow-up^5,20^. In the 891 investigated WHO-confirmed ET patients, the rate of fatal or nonfatal thrombotic events was 1.9% patient–years.^34^ In other studies that included explicitly or a proportion of ET patients diagnosed according to the PVSG criteria,^15^ the corresponding rate ranged from 1.5 to 2.5% patient–years.^35–37^ Therefore, no differences were calculable between the ET or pre-PMF populations regarding thrombosis prior to diagnosis or during follow-up. Of note is that among the other predictors of arterial thrombosis like age >60 years, history of thrombosis, cardiovascular risk factors,^34^ in multivariate analysis leukocytosis was found to be important for ET and pre-PMF as well.^33,36,38^

Concerning history of major bleeding (mostly gastrointestinal) at diagnosis according to the largest cohort of WHO-diagnosed patients until now reported (891 ET and 180 pre-PMF), frequencies were relatively rare (4% vs.7%) and not significantly different.^39^ Contrasting these incidences, major hemorrhage during follow-up occurred only in 6% of ET but in 12% of the pre-PMF patients (*p* = 0.009), consistent with a rate of 0.79 and 1.39% patient–years, respectively (*p* = 0.039).^39^ This result provides persuasive evidence that discrimination of pre-PMF from “true” ET is a significant and independent risk factor for hemorrhagic events. In 311 patients diagnosed with ET according to the PVSG criteria^15^ included in the prospective UK-PT1 trial,^35^ a full range of reticulin scores (four-graded system)^40^ was described, with grades 1 and 2 being particularly frequent, although ~20% of patients had a median reticulin grade of 3.^41^ Increased BM reticulin fibrosis at presentation positively correlated with platelet (*p* = 0.0001) and leukocyte counts WBC (*p* = 0.05) and predicted higher rates of major bleedings during follow-up (HR 2.0; *p* = 0.05), and there was also a strong association between presenting reticulin grade and transformation to MF (HR 5.5; *p* = 0.0007).^41^ In this context, it is tempting to discuss whether those patients are more likely consistent with thrombocythemic pre-PMF than ET.

### Progression to MF and transformation to BP

The clinical impact and the obvious need to discriminate between thrombocythemic pre-PMF and “true” ET is most conspicuously highlighted when regarding outcomes following standardized therapy.^1,2,13^

Progression to overt MF (myelofibrosis with myeloid metaplasia) has been reported according to the previous literature in ET with a cumulative risk at 10 years ranging between 0.8 and 4.5%.^14^ On the other hand, a 10-year cumulative incidence ranging between 12.3%^20^ and 9.7%^5^ in pre-PMF contrasting significantly with a rate of 0.8%^20^ and 0%^5^ for “true” ET, respectively, has been calculated. In a study in ET patients applying diagnostic guidelines in use at the time of first observation (1975–2008) without reclassification the 10-year cumulative incidence of progression to MF was 3.9%.^37^ This high incidence in comparison with strictly WHO-defined ET can be expected because of inclusion with cases diagnosed according to PVSG criteria do not rule out pre-PMF.^15^ In this regard, a comparison of the PVSG diagnostic guidelines^15^ with the British Standard criteria (BCSH)^42^ and the WHO classification^1,2^ is very challenging; alone for the differences in the threshold values for platelets (>600 × 10^9^/L vs. >450 × 10^9^/L). Addressing BM findings as the cornerstone of MPN diagnosis,^26^ in a recent study on 177 low-risk PVSG-defined ET patients, BM specimens were evaluated revealing a surprising 94% concordance with the BCSH guidelines.^43^ This agreement has to be critically reviewed since the PVSG criteria allow less than one-third of the BM biopsy area to show fibrosis without overt MF.^15,41^ In this context, 370 BM biopsies with PVSG-defined ET derived from the UK-PT1 trial^35^ were found by three panelists to present at diagnosis between 37% and 76% fibrosis including higher grades (i.e., grades 3–4)^40^ and also osteosclerosis.^44^ Even more remarkable is the 81% agreement rate between the BCSH guidelines and the 2008 WHO criteria,^1^ however, without any reference to pre-PMF.^43^ Differences between BCSH and WHO criteria including particularly BM morphology regarding accurate ET diagnosis have been previously discussed.^12^

Other large cohorts including two MPN populations documented different follow-ups to death for their WHO-confirmed ET patients (22% vs. 58%) and an overall frequency of developing MF being 9.2% vs. 10.3%, respectively.^25^ In young patients (age ≤ 40 years), fibrotic progression was expectedly higher in ET (16%) for their longer survival.^45^ Risk factors for progression to overt MF include pre-PMF morphology, advanced age, and anemia, whereas the presence of *JAK2*V617F was associated with a lower risk of fibrotic progression.^1^

Transformation to BP (formerly termed acute leukemia-AML) in ET reveals also a wide range according to previous studies with a cumulative risk at 10 years of 0.7–3%.^14^ Following precisely the new WHO criteria a 10-year cumulative incidence was recorded for “true” ET to range between 0.7 and 1.9% (5.20) or was estimated at 12 years to be 1%.^4^ Altogether a risk rate for BP of ~1% at 10 years has been proposed in WHO-diagnosed ET.^26^ Again patients with pre-PMF^5,20^ or failing reclassification of formerly PVSG-defined ET^15^ revealed different incidences at 10 years between 2.3 and 5.8%.^37^ As with fibrotic progression incidence of BP was as high as 2% in younger patients owing to their longer survival.^45^ In a study of over 1100 patients with ET or pre-fibrotic PMF, risk factors for leukemia-free survival were pre-fibrotic PMF, BM morphology, thrombosis, and extreme thrombocytosis (platelets > 1000 × 10^9^/L).^20^

### Prognosis

Applying the WHO diagnostic guidelines,^1–3^ a body of evidence has been produced by several groups that overall median survival in larger cohorts of ET patients ranges from 14.7 to ~21.8 years^18–20,25,46^, significantly different from patients with pre-PMF (ranges 10.5–14.7 years).^4,18–20^ Similar findings for cumulative survival rates were reported.^5,20^ However, far more important is to exclude the influence of age because median survival was 35 years for younger ET patients contrasting 11 years for age groups >60 years.^45^ For this reason, calculation of relative survival rates and loss of life expectancy^18,45,46^ seems to be more appropriate to eliminate the effect of mortality from age-related causes other than the underlying ET on patients’ survival. A comparison between PVSG-versus WHO-confirmed ET reveals a significant loss of life expectancy of 16.5% versus 8.9%^47^ based on the inclusion of pre-PMF in the PVSG classification.^15^ In 891 patients with WHO-defined ET^1,2^ survival was similar to the 2008 Eurostat age- and sex-standardized incidence rates for all causes of death.^20^ Of note, a similar calculation on a cohort of 292 ET patients derived from a single center in the USA revealed a slightly inferior survival rate when compared with a sex- and age-matched population.^25^ Risk factors for overall survival were pre-fibrotic PMF, advanced age, history of thrombosis, leukocytosis, and anemia.^20^

### Driver mutations (*JAK2* V617F/*CALR/MPL* have not been shown to affect survival in ET

Next-generation sequencing (NGS) revealed 53% of 183 patients with WHO-confirmed ET to harbor one or more sequence variants/mutations, other than *JAK2*V617F/*CALR/MPL*; the most frequent were *TET 2* and *ASXL1*.^48^ So-called adverse variants/mutations, in terms of overall, leukemia-free or fibrosis-free survival, included *SH2B3*, *SF3B 1*, *U2AF1*, *TP53*, *IDH 2*, and *EZH 2*; combined prevalence was 15%, respectively. Adverse variants/mutations were associated with inferior survival and the effect was independent of conventional prognostic models; the number of mutations did not provide additional prognostic information.^48^ Moreover, recent observations suggest that women with ET live longer than male patients and that gender may supersede history of thrombosis as a risk variable for overall survival.^49^

Following NGS regarding non-driver mutations patients with pre-PMF showed mutations in *ASXL*1 (18.0%) and *EZH 2* (3.6%); *SRSF* 2 and *IDH* 1/2 mutations were similarly represented.^4^ Noteworthy is that analysis of mutations according to fibrosis grade showed no difference in distribution and allelic burden of driver mutations, whereas any grade of fibrosis was associated with more *ASXL*1 and *EZH* 2 mutations.^4^ Although mutations comprising the high mutation risk category for prognosis or leukemia-free survival (ASXL1, *SRSF* 2, IDH 1/2, EZH 2) were more represented in overt PMF^4,48^, they were also detectable in a pre-PMF (44% vs. 27%, respectively).^4^ In aggregate, it is suggested that patterns of driver and non-driver myeloid gene mutations contribute to prognosis in pre-PMF.

## Different interpretation of risk stratification and therapy

There is general agreement that the primary objective of therapy in ET and thrombocythemic pre-PMF is to prevent fatal thrombohemorrhagic complications.^26,50,51^ In this context as reviewed by Barbui,^50^ the International Prognostic Score for Thrombosis in WHO-ET (IPSET-thrombosis)^52^ was re-analyzed and validated.^53^ Thus, *JAK2*V617F mutation and cardiovascular risk factors allowed the definition of distinct classes of thrombotic risk, including “very low” and “low” risk classes, and between them “intermediate” and “high” risk patients. Validity of the IPSET-thrombosis score in patients with pre-PMF is to be assessed in a well-defined population.

Concerning predictors of survival in pre-PMF, in a multicenter study, stratifying patients by IPSS, the authors failed to detect a significant difference between intermediate-1 and intermediate-2 patients, whereas the high-risk group was clearly distinguishable.^4^ As reviewed by Finazzi et al.,^51^ when taking into account that the majority of patients lie within the lower prognostic IPSS group, observation alone can be recommended.^32,51,54^ On the other hand, patients at intermediate risk may require a symptom-driven treatment for anemia, splenomegaly, or constitutional symptoms. High-risk patients should be treated as overt PMF^32,51,54^.

A recent update on risk-stratification and management of WHO-defined ET recommends also risk-adapted therapy.^26^ Very low-risk ET might not require treatment, whereas aspirin therapy is advised for low-risk disease.^43^ Cytoreductive therapy is recommended for high-risk ET but it is not mandatory for intermediate-risk ET. First-line drug of choice for cytoreductive therapy is hydroxyurea and second-line drugs of choice are interferon-α and busulfan.^26^

The results of two clinical trial support the importance of this distinction, and suggest a re-interpretation of the data. In UK-PT1-randomized clinical trial^35^ it was proved that hydroxyurea was superior to Anagrelide (ANA) in reducing arterial thromboses, particularly, in JAK2-mutated patients; whereas ANA was more efficient in reducing venous thromboses. However, these results did not take into account the distinction between “true” ET and pre-PMF. In contrast, Gisslinger et al.^55^ failed to confirm these results in a randomized clinical trial on patients with confirmed WHO-ET, in which, ANA was not inferior to hydroxyurea in reducing thrombosis. This was attributed to “true” ET having clinical and hematological features different from pre-PMF. Unfortunately, in the ANA arm of the UK-PT1 trial^35^ an excess of MF evolution was shown and this event was confirmed in a large cohort of 3649 high-risk European ET patients.^56^

In conclusion, this review summarizes the most recent changes in ET definition and characteristics following the recent approval of pre-PMF as separate MPN entity. For clinical decision-making, we need a more systematic, controlled, and prospective analysis of the clinical implications of this newly identified variant. A first step would be to launch an expert and critical re-evaluation of cohorts containing old ET and thrombocythemic PMF cases according to the 2016 revised WHO classification, and assess whether the former conclusions, particularly regarding therapy, can be validated. A multicenter prospective study on ET vs. thrombocythemic PMF would be desirable, but its feasibility is questionable.
